# Screening Small Metabolites from Cells as Multifunctional Coatings Simultaneously Improves Nanomaterial Biocompatibility and Functionality

**DOI:** 10.1002/advs.201800341

**Published:** 2018-05-23

**Authors:** Anqi Sun, Zhan Ban, Li Mu, Xiangang Hu

**Affiliations:** ^1^ Key Laboratory of Pollution Processes and Environmental Criteria (Ministry of Education)/Tianjin Key Laboratory of Environmental Remediation and Pollution Control College of Environmental Science and Engineering Nankai University Tianjin 300071 China; ^2^ Tianjin Key Laboratory of Agro‐environment and Safe‐product Key Laboratory for Environmental Factors Control of Agro‐product Quality Safety (Ministry of Agriculture) Institute of Agro‐environmental Protection Ministry of Agriculture Tianjin 300191 China; ^3^ Key Laboratory of Pollution Processes and Environmental Criteria (Ministry of Education)/Tianjin Key Laboratory of Environmental Remediation and Pollution Control College of Environmental Science and Engineering Nankai University Tianjin 300071 China

**Keywords:** antibacterial materials, biocompatibility, metabolomics, nanoparticles, nanosilver

## Abstract

Currently, nanomaterials face a dilemma due to their advantageous properties and potential risks to human health. Here, a strategy to improve both nanomaterial biocompatibility and functionality is established by screening small metabolites from cells as nanomaterial coatings. A metabolomics analysis of cells exposed to nanosilver (nAg) integrates volcano plots (*t*‐tests and fold change analysis), partial least squares‐discriminant analysis (PLS‐DA), and significance analysis of microarrays (SAM) and identifies six metabolites (l‐aspartic acid, l‐malic acid, myoinositol, d‐sorbitol, citric acid, and l‐cysteine). The further analysis of cell viability, oxidative stress, and cell apoptosis reveals that d‐sorbitol markedly reduces nAg cytotoxicity. Subsequently, small molecule loading, surface oxidation, and ionic release experiments support d‐sorbitol as the optimal coating for nAg. Importantly, d‐sorbitol loading improves the duration of the antibacterial activity of nAg against *Escherichia coli* and *Staphylococcus aureus*. The biocidal persistence of nAg‐sorbitol is extended beyond 9 h, and the biocidal effects at 12 h are significantly higher than those of naked nAg. This work proposes a new strategy to improve the biocompatibility and functionality of nAg simultaneously by screening small metabolites from cells as nanomaterial functional coatings, a method that can be applied to mitigate the side effects of other nanomaterials.

## 1 Introduction

Nanomaterials have been widely applied in various fields due to their advantageous mechanical, electronic, optical, physiochemical, and physicochemical properties.^1^, ^2^, ^3^ For example, there are more than 2000 nanomaterial‐based products, of which over 25% include Ag.^4^ Nanosilver (nAg) has been widely used for biocidal purposes (e.g., in medical devices, food storage, cosmetics, textiles, and pigments) for over a century.^5^, ^6^, ^7^ However, the health risks and adverse effects (e.g., nanotoxicity in vivo and in vitro) of nanomaterials caused by their high specific surface area and physiochemical activity are also attracting increasing attention.^8^, ^9^, ^10^, ^11^ Many methods have been proposed to reduce nanotoxicity, such as surface modification and the regulation of shapes, sizes, charges, defects, and crystal faces.^12^, ^13^, ^14^, ^15^ Unfortunately, the resulting improvements in the biocompatibility of nanomaterials were in most cases accompanied by reduced functionality. For instance, chemical modifications would reduce photocatalytic activity. Therefore, a universal strategy to improve nanomaterial biocompatibility and functionality simultaneously is urgently needed, as both properties determine the ability to commercialize nanomaterials.^16^, ^17^, ^18^

The uncontrolled release of Ag^+^ from the oxidized surface of nAg (Ag_2_O+H_2_O→2Ag^+^+2OH^−^) exerts hazardous effects on organisms and limits the widespread use of nAg in healthcare.^19^, ^20^, ^21^, ^22^, ^23^, ^24^, ^25^ To hinder ionic release and prevent the access of oxygen to the nAg surface, surface coatings are expected to be a useful tool.^26^, ^27^ For example, the Ag^+^ on the surface of nAg was found to preferentially bind cysteine to form a nAg‐cysteine complex, which was biocompatible and stable in vivo.^28^, ^29^ However, most chemical modifications are accompanied by weakening of the antibacterial ability of nAg.^30^, ^31^, ^32^ Hence, constructing an oxygen‐isolation layer without damaging the original biocidal properties of nAg is of vital importance.

To avoid altering the advantages of nanomaterials, modification with small molecules is likely to be a better choice than modification with proteins, DNA or polymers, although there is no golden rule for the screening of ideal molecules. Moreover, natural molecules are generally more biocompatible than synthetic molecules. Metabolites are natural molecules that are widely distributed in the human body. Metabolites that are downregulated by nanomaterials indicate the targets of the nanomaterials.^33^ Cysteine is a well‐known biological target of nAg and the decoration of cysteine reduces the nanotoxicity of nAg.^34^, ^35^ Therefore, we supposed that exogenous supplementation of downregulated metabolites or the use of downregulated metabolites as nanomaterial coatings could alleviate the interaction of nAg on intracellular metabolites and thus mitigate nanotoxicity. High‐throughput metabolomics is an untargeted trial and can comprehensively reveal changes of metabolic profiles.^36^, ^37^ Inspired by metabolomics, this work provides a strategy to improve nanomaterial biocompatibility and functionality simultaneously by screening small metabolites from cells as nanomaterial functional coatings.

First, the metabolites downregulated by nanomaterials were identified by metabolomics screening. Then, each screened metabolite was tested for the effective mitigation of nanotoxicity. Meanwhile, the effects of the screened metabolites on nanomaterial stability were analyzed to obtain stable nanomaterial‐metabolite complexes. The metabolite with the most positive effects on nanotoxicity (e.g., cytotoxicity analysis) was further screened, and its specific detoxification mechanisms were explored (e.g., decreased intracellular Ag^+^ release from nAg). Subsequently, the nanomaterials were coated with the best metabolite identified to confirm the mitigation of cytotoxicity. Finally, the enhanced functions (e.g., the antibacterial effect of nAg on *Escherichia coli* and *Staphylococcus aureus*) of the coated nanomaterials were compared with those of the naked nanomaterials. The above strategy successfully obtained the best metabolite coating for nanoparticles, thereby simultaneously improving nanomaterial biocompatibility and functionality.

## 2 Results and Discussion

### 2.1 Screening Ideal Metabolites to Control Nanotoxicity

**Figure 1:**
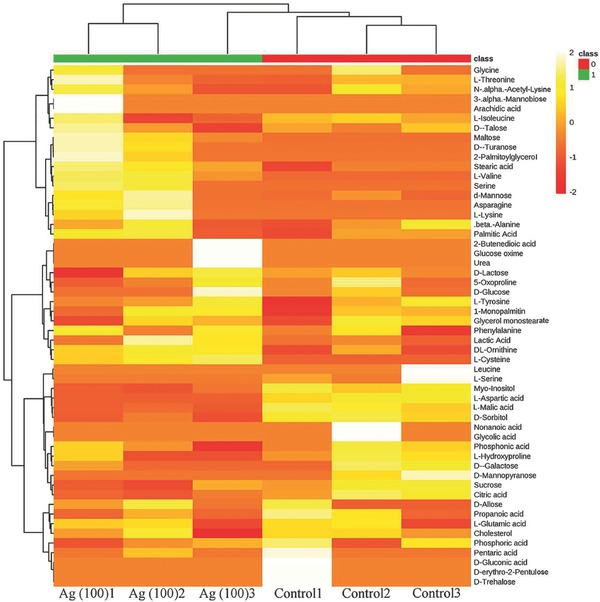
Heat map of metabolites detected by GC‐MS. “Ag (100)” indicates groups treated with nAg at 100 mg L^−1^.

**Figure 2:**
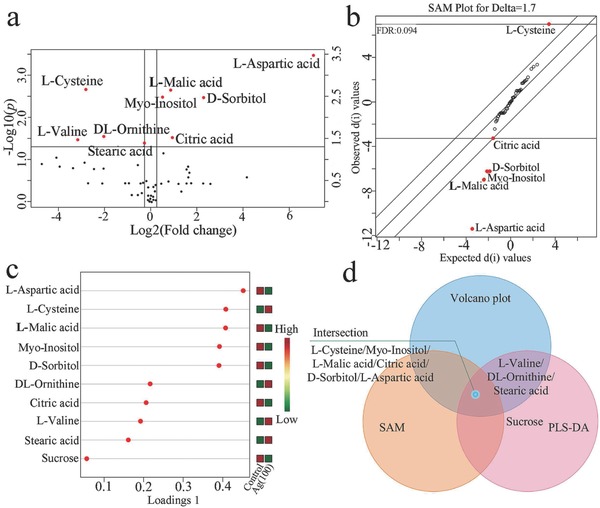
Metabolomics screening for downregulated metabolites in cells exposed to nAg: a) Nine metabolites were identified by volcano plot (fold change >1.4 and *p* < 0.05); b) six metabolites were screened by SAM (SAM plot for delta = 1.7); c) ten metabolites were screened by PLS‐DA according to the variable importance in projection scores; d) Venn diagram of the screened metabolites.

The nAg we used had spherical and angular shapes, and the size distribution ranged from ≈20 to 160 nm with most of the nanoparticles concentrated in the range 60–80 nm (Figure S1, Supporting Information). The size of used nAg was homogeneous and consistent with the reported nAg in industrial applications.^38^ Size distribution is an important property of nanomaterials,^39^ and the effects of sizes on nanotoxicity have been widely discussed.^38^ The present work verified the obvious toxicity of used nAg, and focused on the control of nanotoxicity by surface function. As shown in Figure S2 (Supporting Information), 100 mg L^−1^ nAg significantly decreased the cell viability of LO_2_ to 72.03 ± 15.18% (*p* < 0.01). The nanotoxicity control experiments tested the metabolic toxicity of nAg at 100 mg L^−1^. Metabolomics analysis identified a total of 54 metabolites from ≈250 peaks for each group (**Figure** **1**). The identified metabolites included fatty acids, amino acids, carbohydrates, and other biomolecules. Metabolomics analysis is an untargeted and random screening trial, and the results usually exhibit a large standard deviation. To obtain the ideal metabolites for the control of nanotoxicity, volcano plots (*t*‐tests *p* < 0.05 and fold change >1.4), partial least squares‐discriminant analysis (PLS‐DA, top 10 according to the variable importance in projection scores) and significance analysis of microarrays (SAM, delta = 1.7) were used to examine the 54 identified metabolites. Nine, six, and ten metabolites were selected by the above three models, respectively, as shown in **Figure** **2** and Tables S1–S3 (Supporting Information). Furthermore, six metabolites (i.e., l‐aspartic acid, l‐malic acid, myoinositol, d‐sorbitol, citric acid, and l‐cysteine) were found in all three statistical analyses, as shown in Figure 2d and Table S4 (Supporting Information). The five metabolites other than l‐cysteine were downregulated by nAg compared with the control. l‐cysteine has been reported to moderate the nanotoxicity of nAg by complexing the released Ag^+^.^34^ The upregulation of l‐cysteine could be a self‐defense approach toward nAg invasion. Until now, no studies have focused on the detoxification effects of the other five metabolites downregulated by nAg. l‐cysteine and the other optimized metabolites will be compared in the following sections to address the advantages of the optimized metabolites. The addition of the downregulated metabolites may recover the imbalance caused by nAg. Since the ingestion of excessive metabolites can cause metabolic dysfunction,^40^ the contents of the downregulated metabolites were quantified prior to their addition. The appropriate concentrations of l‐aspartic acid, citric acid, l‐malic acid, d‐sorbitol, and myoinositol were ≈200, 16, 11, 77, and 4.5 ng per 10^6^ cells, respectively (Table S5, Supporting Information).

### 2.2 Metabolites Mitigate the Cytotoxicity of nAg

**Figure 3:**
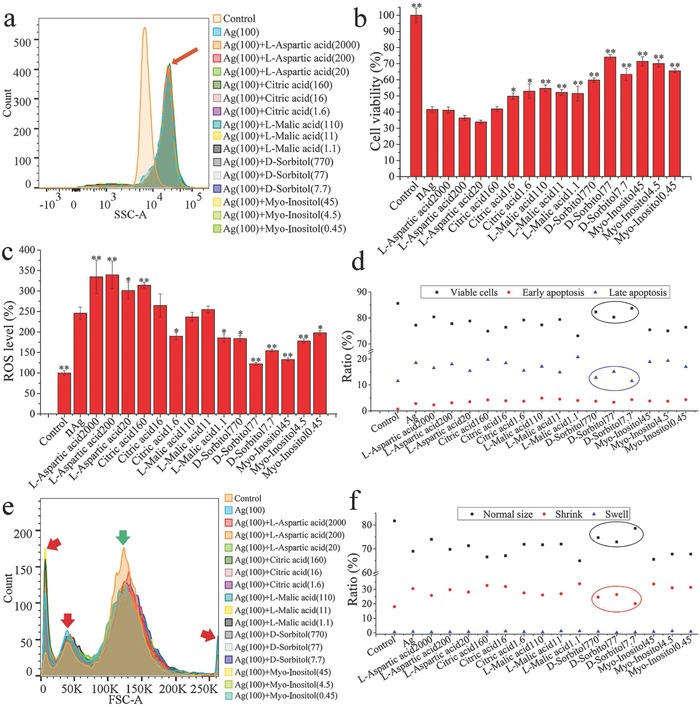
Screened metabolites mitigate the cytotoxicity caused by nAg: a) Cell uptake of nAg; b) cell viability; c) reactive oxygen species (ROS) in cells; d) cell apoptosis; e) cell with abnormal sizes induced by nAg; f) change in cell size. “Ag (100)” in panels (a) and (e) indicates groups treated with nAg at 100 mg L^−1^. The red arrow in panel (a) denotes the similar uptake potential of nAg with or without metabolite treated. The numbers after the metabolite names are the optimized concentrations of the added metabolites (ng per 10^6^ cells). “*” and “**” indicate *p* < 0.05 and *p* < 0.01, respectively, compared with the values in cells treated with 100 mg L^−1^ nAg. The black and blue circles in panel (d) denote cells treated with d‐sorbitol in the normal state and in late apoptosis, respectively. The green arrow in panel (e) denotes the normal cells (as cells in the control), and the red arrows denote abnormal cells. The black and red circles in panel (f) denote cells treated with d‐sorbitol at normal and shrunken sizes, respectively.

The intensity of side scatter (SSC) in flow cytometer (FCM) is positively correlated with the particle uptake.^41^ The intensity of SSC increased in the nAg‐treated groups compared with the control, as denoted by the red arrow in **Figure** **3**a. The overlapping peaks (denoted by the red arrow) indicated that the uptake of nAg remained similar with and without metabolite treatment, suggesting that the added metabolites did not directly affect nAg uptake. Figure 3b shows that the added metabolites (citric acid, l‐malic acid, d‐sorbitol, and myoinositol) markedly mitigated the reduction in cell viability induced by nAg (*p* < 0.05), especially d‐sorbitol and myoinositol (*p* < 0.01). The cell viability recovered to over 74% and 71% with the addition of d‐sorbitol and myoinositol, respectively. The cell viability recovered to over 52% and 54% with the addition of citric acid and l‐malic acid, respectively. Excess reactive oxygen species (ROS) leads to mitochondrial dysfunction, which in turn results in ROS formation and cell apoptosis.^42^ As shown in Figure 3c, the increase in ROS level caused by nAg was reduced by the addition of the above four metabolites, especially for d‐sorbitol and myoinositol (*p* < 0.05). The ROS increase induced by nAg was reduced by 50.2% and 46.1% by d‐sorbitol and myoinositol, respectively. In contrast, citric acid and l‐malic acid inhibited the ROS increase by only 22.9% and 24.5%, respectively. The data from the cell apoptosis tests (Figure 3d; Figure S3, Supporting Information) confirmed the protective effects of d‐sorbitol on nAg‐induced apoptosis. The proportion of cells in the late apoptosis phase was reduced by 18.38–37.84% by d‐sorbitol, as denoted by the blue circle in Figure 3d. The proportion of intact cells in the d‐sorbitol‐treated groups was 4.02–8.42% higher than that in the nAg‐treated group, as circled by the black line. The reduction of cells in the early apoptosis phase induced by d‐sorbitol did not show even a slight increase, indicating that d‐sorbitol balanced the programmed apoptosis and inhibited the transition of cells from early apoptosis to late apoptosis. The intensity of forward scatter (FSC) in FCM is proportional to the cell size, and cell shrinkage or swelling reflected cell death.^43^ As shown in Figure 3e (the green and red arrows), nAg decreased the counts of normal‐sized cells and increased the counts of abnormal‐sized cells. The proportion of cells with normal size in the d‐sorbitol‐treated groups (72.9–78.6%) was higher than in the nAg‐treated groups (69%), as shown in Figure 3f and Figure S4 (Supporting Information). Unlike the other metabolites, d‐sorbitol maintained the normal cell size and reduced the proportion of abnormal cells by 13.16–33.88%, especially shrunken cells and apoptosis bodies in the nAg‐treated groups, as denoted by the red circles in Figure 3f. Cells in the late apoptosis period underwent disassembly into subcellular fragments,^44^ and thus, the decrease in the number of small cells was consistent with the decrease in the number of cells in the late apoptosis period (Figure 3d,f). Collectively, the screened metabolites exhibited clear potential to mitigate the cytotoxicity caused by nAg, and the protective effects of d‐sorbitol were more obvious than those of the rest. In most previous studies, the coatings for mitigating nanotoxicity were chosen based on experience.^45^ This study is the first to build a metabolomics‐based strategy to screen for small molecules that reduce nanotoxicity.

### 2.3 High Stability of nAg/d‐Sorbitol Complex

**Figure 4:**
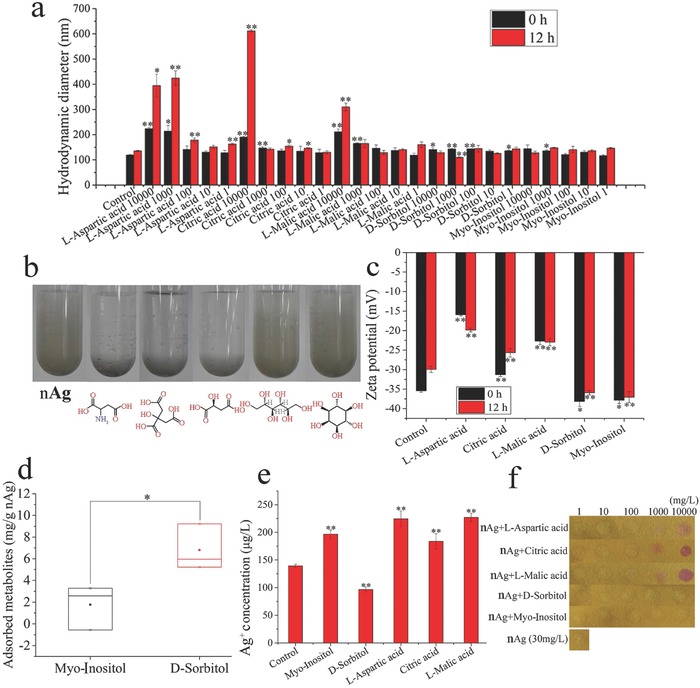
Dispersity and Ag^+^ release of naked nAg and nAg/metabolite complexes: a) Hydrodynamic diameters of nAg and nAg/metabolite complexes; b) digital images of nAg and nAg/metabolite complexes at 10 000 mg L^−1^; c) zeta potential of nAg and nAg/metabolite complexes at 100 mg L^−1^; d) adsorption of myoinositol and d‐sorbitol on nAg; e) quantification of Ag^+^ release from nAg and nAg/metabolite complexes after 12 h; f) pH values of naked nAg and nAg/metabolite complex suspensions. “**” in panels (a) and (c) indicates *p* < 0.01 compared with 30 mg L^−1^ nAg at the initial time, while “##” indicates *p* < 0.01 compared with 30 mg L^−1^ nAg after 12 h. “*” in panel (d) indicates *p* < 0.05. “**” in panel (e) indicates *p* < 0.01 compared with 30 mg L^−1^ nAg. The numbers after the metabolite names are the concentrations (mg L^−1^) of the added screened metabolites.

The transformation and agglomeration of nAg determine its speciation and bioavailability.^46^ The addition of the metabolites (i.e., citric acid, l‐malic acid, and l‐aspartic acid) significantly increased the hydrodynamic diameter of nAg from ≈100 nm (diameter measured by electron microscopy were 60–80 nm) to 200 nm (**Figure** **4**a). In addition, the increase in hydrodynamic diameter grew from 0 to 12 h. The aggregation of nAg is also verified by the digital images in Figure 4b. The hydrodynamic diameter of nAg was increased by d‐sorbitol, but the slight increase was not enhanced over time (Figure 4a). d‐sorbitol at 1 mg L^−1^ increased the hydrodynamic diameter of nAg from 119.03 ± 4.96 to 135.73 ± 4.07 nm (*p* < 0.05) at 0 h. In contrast, myoinositol at 1 mg L^−1^ did not increase the hydrodynamic diameters of nAg (Figure 4a). The increase of diameters of nanomaterials was due to the formation of surface coatings.^47^ Therefore, d‐sorbitol showed a better wrapping effect onto nAg at low concentration (1 mg L^−1^) compared with myoinositol. The zeta potential of nAg increased from −35 mV to above −30, −22, and −20 mV after the addition of citric acid, l‐malic acid and l‐aspartic acid, respectively (Figure 4c), implying that the aggregation of nAg increased. In contrast, d‐sorbitol and myoinositol (100 mg L^−1^) decreased the zeta potential of nAg to less than −35 mV, suggesting that these two molecules enhanced the stability of nAg. Moreover, Figure 4d illustrated that more d‐sorbitol (over 6 mg per g nAg) than myoinositol (below 2 mg per g nAg) adsorbed onto nAg. The differences in the adsorption of these two isomers on nAg can probably be attributed to their ring (d‐sorbitol) and linear (myoinositol) structures.^48^

Furthermore, the amount of Ag^+^ released from the nAg‐metabolite complex was determined. As shown in Figure 4e, only d‐sorbitol decreased the ion release from nAg, from 139.24 ± 4.99 to 96.63 ± 7.61 µg L^−1^ (*p* < 0.01). The other four metabolites did not inhibit Ag^+^ release and even increased it to greater than 170 µg L^−1^ (*p* < 0.01, Figure 4e). In the presence of Ag^+^ on the nAg surface, the zeta potential became less negative owing to the offset of electrons.^49^ The Ag^+^ release results were consistent with the zeta potentials shown in Figure 4c. Since both protons and dissolved oxygen participate in the oxidation of nAg,^50^, ^51^ released Ag^+^ increases with decreasing pH. In contrast to d‐sorbitol and myoinositol, the metabolites l‐aspartic acid, citric acid, and l‐malic acid clearly decreased the medium pH (Figure 4f). Unlike d‐sorbitol, the presence of myoinositol did not decrease the level of Ag^+^ release, possibly owing to the low adsorption of myoinositol onto nAg, as shown in Figure 4d. Overall, d‐sorbitol protected nAg from aggregation and transformation, particularly the release of Ag^+^, which was linked to cytotoxicity.

### 2.4 d‐Sorbitol Inhibits Ag^+^ Release from Intracellular nAg by Surface Passivation

**Figure 5:**
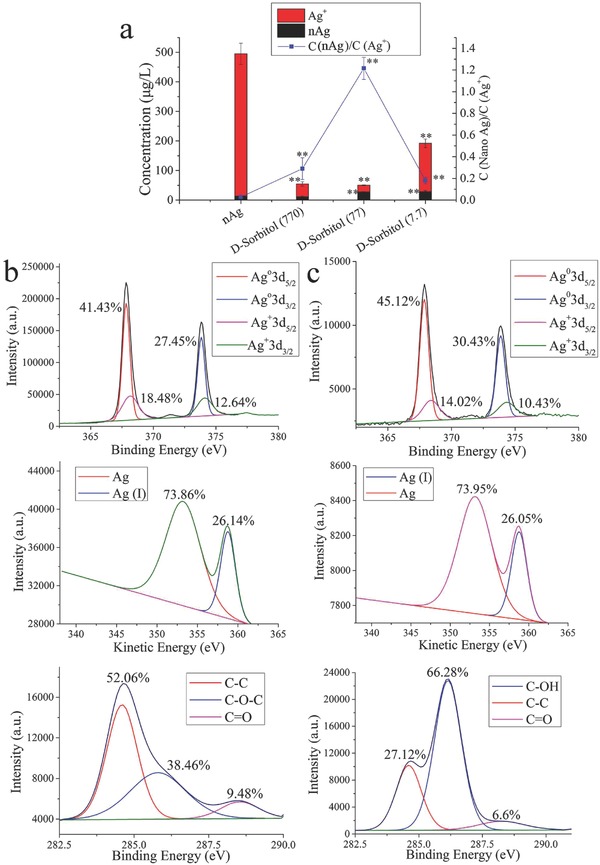
d‐sorbitol inhibits Ag^+^ release in cells: a) Quantification of nAg and Ag^+^ in cells; b) XPS spectra of nAg; c) XPS spectra of nAg‐sorbitol complex. “**” in panel (a) means *p* < 0.01 compared with the nAg‐treated cells. “C” in the legend of panel (a) means concentration.

To confirm the inhibitory effect of d‐sorbitol on Ag^+^ release from nAg in cells, the intracellular nAg and Ag^+^ were quantified. As shown in **Figure** **5**a, the concentration ratio of nAg to Ag^+^ was enhanced from 0.03 to over 0.29 by the addition of d‐sorbitol. However, d‐sorbitol did not markedly decrease the concentration of nAg, suggesting that the effects of d‐sorbitol relied on the suppression of Ag^+^ release rather than the exocytosis of nAg. Furthermore, the surface passivation of nAg complexed with d‐sorbitol was confirmed by X‐ray photoelectron spectroscopy (XPS). The Ag 3d spectrum showed four peaks, Ag^0^3d_5/2_, Ag^0^3d_3/2_, Ag^+^3d_5/2_, and Ag^+^3d_3/2_,^52^ as shown in Figure 5b,c. The presence of Ag^+^3d_5/2_ and Ag^+^3d_3/2_ indicated the oxidation of nAg.^53^ The percentages of Ag^+^3d_5/2_/Ag^+^3d_3/2_ in naked nAg and in nAg complexed with d‐sorbitol were 18.48%/12.64% and 14.02%/10.43%, respectively, which suggested that d‐sorbitol passivated the nAg surface, protecting it from oxidation. The Ag(0)/Ag(I) ratio in the Ag‐Auger spectrum also increased from 2.82 to 2.84 after d‐sorbitol treatment. The above results confirmed the surface passivation (inhibiting oxidation) of nAg by the addition of d‐sorbitol, which then inhibited Ag^+^ release and further cytotoxicity, as shown in Figure 3.

In this work, Ag^+^ from AgNO_3_ was used as a negative control. The Ag^+^‐decreased cell viability and Ag^+^‐increased ROS level were not inhibited by d‐sorbitol, as shown in Figure S5a,b (Supporting Information). No reduction of Ag^+^ was observed upon quantifying Ag^+^ concentration in the AgNO_3_‐sorbitol solutions (Figure S5c, Supporting Information). Therefore, the positive effects of d‐sorbitol on nAg‐induced cytotoxicity are dependent on the surface passivation of AgNPs rather than a direct influence on Ag^+^. Furthermore, the contents of d‐sorbitol from cells treated with nAg decorated d‐sorbitol were analyzed and did not significantly increase (Figure S6, Supporting Information). Thus, decoration of d‐sorbitol on nAg was stable in cells. Moreover, l‐aspartic acid and l‐malic acid were simultaneous upregulated by the addition of d‐sorbitol (Figure S6, Supporting Information). l‐aspartic acid and l‐malic acid played key roles on cellular function control and repair,^54^, ^55^ which could be the indirect impacts of d‐sorbitol on the mitigation of nanotoxicity.

### 2.5 Loading d‐Sorbitol Enhances nAg Dispersity and Maintains the Native Shape of nAg

**Figure 6:**
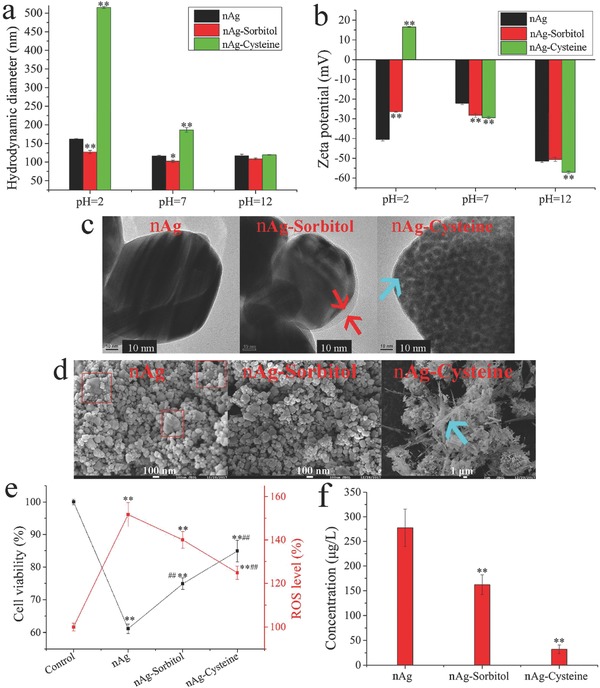
Characterization of nAg, nAg‐sorbitol, and nAg‐cysteine, with comparisons of nanomaterial biocompatibility: a) Hydrodynamic diameters of synthesized nAg‐based nanomaterials; b) zeta potential of synthesized nAg‐based nanomaterials; c) TEM images of synthesized nAg‐based nanomaterials; d) SEM images of synthesized nAg‐based nanomaterials; e) cell viability and ROS levels in cells treated with nAg, nAg‐sorbitol, and nAg‐cysteine; f) Ag^+^ release from nAg, nAg‐sorbitol, and nAg‐cysteine. “**” in panels (a), (b), and (f) indicates *p* < 0.01 compared with nAg. “**” in panel (e) indicates *p* < 0.01 compared with cells in the control groups, and “##” indicates *p* < 0.01 compared with the cells treated with nAg. The red and blue arrows in panel (c) denote the transparent layers formed by d‐sorbitol and Ag_2_S, respectively. The red boxes and the blue arrows in panel (d) indicate large nAg and Ag nanoneedles, respectively.

nAg loaded with cysteine was used as a positive control due to the well‐known ability of cysteine to reduce Ag^+^ back to Ag^0^.^34^ The synthesized Ag‐based nanomaterials are shown in Figure S7 (Supporting Information). nAg‐sorbitol exhibited higher dispersity than naked nAg or nAg‐cysteine. As shown in Figure S8 (Supporting Information), the size distribution of nAg‐sorbitol was narrow (20–110 nm) compared with that of nAg (20–160 nm) and nAg‐cysteine (0–140 nm). As shown in **Figure** **6**a, nAg loaded with d‐sorbitol exhibited smaller hydrodynamic diameters than naked nAg (127.1 ± 7.1 vs 161.7 ± 1.9 nm at pH 2, 102.6 ± 4.6 vs 116.5 ± 3.6 nm at pH 7, and 108.9 ± 3.9 vs 117.1 ± 6.6 nm at pH 12, respectively). The size of nAg‐sorbitol showed a slight decrease with increasing pH; the diameter was higher in an acid environment (127.1 ± 7.1 nm) than in neutral (102.6 ± 4.5 nm) and alkaline (108.9 ± 3.9 nm) environments, indicating the binding of H^+^ to the hydroxyls of d‐sorbitol. The zeta potential of nAg‐sorbitol (−28.2 ± 2.1 mV) was lower than that of nAg (−22.1 ± 1.5 mV) in a neutral environment (Figure 6b). The decrease in zeta potential suggested an increase in electronegative groups,^35^ that is, the coating of hydroxyls from d‐sorbitol. In contrast, the presence of H^+^ shielded the electronegativity of the hydroxyls, as shown by the higher zeta potential in an acid environment (−26.4 ± 0.5 mV) than in a neutral environment (−28.2 ± 2.1 mV). Owing to the complexation of Ag^+^ by sulfydryls from l‐cysteine,^56^ the hydrodynamic diameter of nAg coated with l‐cysteine at pH 7 (186.3 ± 10.6 nm) was higher than that of naked nAg (116.5 ± 3.6 nm). The uniform black dots formed on the surface of nAg‐cysteine, indicated by the blue arrow in Figure 6c confirmed Ag_2_S complexation. In contrast to naked nAg and nAg‐cysteine, no large aggregates (red boxes in Figure 6d) were found in the nAg‐sorbitol complex. Except for a thin translucency (thickness less than 10 nm) formed around the nAg (red arrows in Figure 6c), the sorbitol coating did not change the spherical and angular shapes of nAg. In contrast, l‐cysteine changed the morphology of Ag nanoparticles to Ag needles (Figure 6d). The distribution of S was consistent with the formation of the Ag needles, as indicated by the blue boxes in Figure S9 (Supporting Information). This reconfiguration of nAg occurred because of the bridging of nanoparticles with l‐cysteine.^46^ The above results supported that loading d‐sorbitol enhanced nAg dispersity and maintained the native shape of nAg, in contrast to the well‐known l‐cysteine modification.

### 2.6 Loading d‐Sorbitol Mitigates nAg Nanotoxicity

As shown in Figure 6e, the cell viability was lower than 60%, over 75% and over 80% after treatment with naked nAg, nAg‐sorbitol, and nAg‐cysteine, respectively. Compared with the control, the enhanced ROS levels produced by naked nAg, nAg‐sorbitol, and nAg‐cysteine were over 150%, less than 140%, and less than 130%, respectively. The electronegative groups on the surface of nAg could inhibit the oxidation of nAg and were available for the complexation of released Ag^+^.^15^ Compared with the amount of Ag^+^ released by naked nAg (277.6 ± 65.7 µg L^−1^), the nAg‐sorbitol (162.1 ± 34.5 µg L^−1^), and nAg‐cysteine (132.1 ± 15.2 µg L^−1^) groups both released less (Figure 6f). The inhibition of Ag^+^ release contributed to the mitigation of nanotoxicity. The XPS data showed that the ratios of lattice oxygen/adsorbed oxygen in nAg‐sorbitol, nAg‐cysteine, and naked nAg were 0.71, 2.55, and 0.35, respectively (Figure S10, Supporting Information). The functional groups coated onto nAg sequestered oxygen from the air.^57^ Therefore, less adsorbed oxygen could be detected on the surface of nAg wrapped by d‐sorbitol or l‐cysteine. The Ag^+^3d/Ag^0^3d ratios in naked nAg, nAg‐cysteine, and nAg‐sorbitol were 0.50, 2.24, and 0.45 (Figure S10, Supporting Information). This result was consistent with the lower ratio of Ag(I)/Ag(0) in the Ag‐Auger spectrum of nAg‐sorbitol (0.25) than that of naked nAg (0.26). The high proportion of Ag^+^3d found in nAg‐cysteine was due to the formation of Ag_2_S rather than oxidized nAg, as indicated by the low ratio (0.18) of Ag(I)/Ag(0) in the Ag‐Auger spectrum. Therefore, the high proportions of lattice oxygen in nAg‐sorbitol and nAg‐cysteine were not related to nAg oxidation but to the O atoms in d‐sorbitol and l‐cysteine. Furthermore, few S atoms and little Ag‐S were found in nAg‐sorbitol (Figure S10, Supporting Information), and they probably originated from the air. In addition to the stabilizing effects of d‐sorbitol on nAg, the presence of Ag‐S might also alleviate the nanotoxicity. Although nAg‐sorbitol and nAg‐cysteine showed comparable biochemistry, d‐sorbitol enhanced the dispersity of nAg more strongly, and maintained the native shape of nAg better than l‐cysteine, as shown by the results and discussion above. The high dispersity and the native shape of nAg are critical to its applications.^58^ Collectively, the coating of d‐sorbitol could reduce the nanotoxicity of nAg and simultaneously maintain or even improve its intended effects, as confirmed below.

### 2.7 High and Lasting Antibacterial Activity of d‐Sorbitol‐Loaded nAg

**Figure 7:**
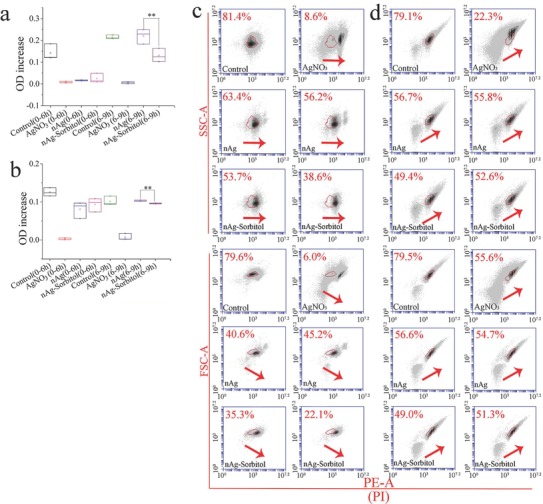
Antibacterial ability of Ag^+^ and nAg‐based nanomaterials: a) Ag‐induced time‐dependent OD changes of *E. coli*; b) Ag‐induced time‐dependent OD changes of *S. aureus*; c) biocidal effects on *E. coli*; d) biocidal effects on *S. aureus*. The red arrows denote the shift tendency of the cell population. “**” in panels (a) and (b) suggests *p* < 0.01 compared with bacteria treated with nAg.

The antibacterial effect of nAg was largely dependent on the Ag^+^ release.^59^ l‐cysteine‐loaded nAg exhibited reduced nanotoxicity but also low antibacterial activity.^59^ Unlike sulfhydryl groups, hydroxyl groups bind Ag^+^ weakly.^60^ d‐sorbitol loading was established to improve the biocompatibility of nAg, and then the nanomaterial function (antibacterial ability) was investigated. As shown in **Figure** **7**, the presence of AgNO_3_ (100 mg L^−1^) sustainably inhibited bacterial proliferation at 6 and 9 h, with no obvious optical density (OD) increase for either *Escherichia coli* (*E. coli*, Gram‐negative bacteria, G^−^) or *Staphylococcus aureus* (*S. aureus*, Gram‐positive bacteria, G^+^). Similar biocidal effects on *E. coli* were observed for 200 mg L^−1^ nAg and 200 mg L^−1^ nAg‐sorbitol at 6 h; however, the proliferation of *E. coli* cultured with naked nAg recovered to the baseline level at 9 h. The increase of OD values (6–9 h) in the control and nAg‐treated groups were 0.215 ± 0.009 and 0.220 ± 0.034, respectively, demonstrating the loss of antibacterial activity. In contrast, the OD value increase (6–9 h) in the nAg‐sorbitol group was 0.130 ± 0.031, demonstrating that the antibacterial effects against *E. coli* were persistent. Higher antibacterial activity against *S. aureus* was also observed at 9 h for nAg‐sorbitol than for naked nAg (*p* < 0.01, Figure 7b), although there were no significant differences in biocidal ability between the two nanomaterials during the first 6 h. If d‐sorbitol at 1000 mg L^−1^ and nAg were added separately, obviously antibacterial effect on *E. coli* was found compared to nAg (*p* < 0.01, Figure S11a, Supporting Information). The outstanding antibacterial ability was endowed by the biocidal effects of d‐sorbitol at initial 12 h (*p* < 0.01, Figure S11a, Supporting Information). As for *S. aureus*, though d‐sorbitol at 1000 mg L^−1^ exhibited significant antibacterial ability during the initial 12 h, the addition of d‐sorbitol did not enhance the original biocidal effects of nAg (Figure S11b, Supporting Information). In contrast, nAg‐sorbitol at 200 mg L^−1^ with 0.5% d‐sorbitol (approximately equal to 1 mg L^−1^, measured from the adsorption capacity, 6 mg per g nAg in the Section 2.3) exhibited higher antibacterial effects on both of *E. coli* and *S. aureus* than separately added d‐sorbitol (over 1 mg L^−1^) and nAg (200 mg L^−1^). In 12–24 h, the antibacterial ability of d‐sorbitol even disappeared on *S. aureus*, however, the biocidal effects of nAg‐sorbitol were still stronger than nAg (*p* < 0.01). Therefore, the antibacterial capacity of nAg‐sorbitol complex was better than that of separately adding nAg and d‐sorbitol. The proton motive force of the bacteria decreased the local pH of the cytoplasm and membrane,^61^ thus promoting the release of Ag^+^ from nAg‐sorbitol. Unlike that of naked nAg, the zeta potential of nAg‐sorbitol increased in an acid environment (Figure 6b). The zeta potential of naked nAg decreased from approximately −20 to −40 mV as the pH decreased. In contrast, the zeta potential of nAg‐sorbitol was −28.24 ± 2.15 mV in a neutral environment and −26.44 ± 0.46 mV in an acid environment. The particle instability of nAg was accompanied by Ag^+^ release,^62^ which resulted in the long‐lasting Ag^+^ release of nAg‐sorbitol in a local acid environment. Thus, the antimicrobial effects of nAg‐sorbitol were more persistent than those of naked nAg against both G^−^ and G^+^.

The cytometric analysis further confirmed the above results, as shown in Figure 7c,d and Figure S12 (Supporting Information). After nAg treatment, the bacterial population of *E. coli* was right shifted owing to the increase in propidium iodide (PI) stained dead cells (Figure 7c; Figure S12a, Supporting Information). The percentages of bacteria unstained with PI were 46.15 ± 10.68% and 59.80 ± 5.09% in the groups treated with nAg‐sorbitol and naked nAg, respectively. The obvious decrease in FSC signals in the nAg‐sorbitol treated groups (28.70 ± 9.33%) compared with the naked nAg‐treated groups (42.90 ± 3.25%) also suggested that the d‐sorbitol coating enhanced the antibacterial effects. The bacterial population of *S. aureus* was upward shifted with a slight right shift (Figure 7d; Figure S12b, Supporting Information). The strong SSC signals indicated ripening cells with high intracellular granularity and were simultaneously associated with the increased FSC signals.^63^ As bactericides, nAg disrupted the cell wall integrity of G^−^ and inhibited the cell division of G^+^.^64^ Therefore, *S. aureus* treated with nAg accumulated in the ripening phase rather than the growth phase, as indicated by the upward shifts of both SSC and FSC. Herein, the percentages of bacteria in the growth phase after culturing with nAg‐sorbitol and naked nAg were 51 ± 2.26% and 56.25 ± 0.64% in SSC‐PI analysis and 50.15 ± 1.63% and 55.65 ± 1.34% in FSC‐PI analysis, respectively (Figure 7d; Figure S12b, Supporting Information). Collectively, nAg‐sorbitol exhibited higher biocompatibility and more lasting antibacterial ability than naked nAg.

## 3 Conclusion

**Figure 8:**
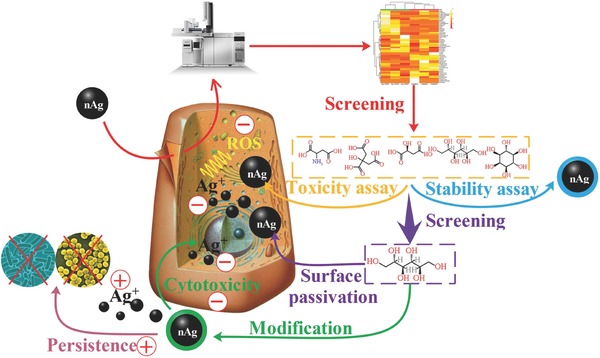
Metabolomics‐screened d‐sorbitol as a surface coating reduced cytotoxicity and enhanced antibacterial ability of nAg. The plus signs indicate positive effects while the minus signs indicate negative effects.

Nanotoxicity hinders the effective application of nanomaterials. Many methods have been proposed to reduce nanotoxicity, but in most cases, the improvements in biocompatibility reduce the functionality of the nanomaterials. The establishment of a strategy to improve nanomaterial biocompatibility and functionality simultaneously is essential, as both properties determine the ability to commercialize nanomaterials. This work is the first to establish a strategy of using metabolomics to screen for small molecules that can simultaneously reduce nanotoxicity and enhance nanomaterial functionality, as shown in **Figure** **8**. The screened d‐sorbitol protected nAg from aggregation and transformation, particularly inhibiting the release of Ag^+^, which is linked to cytotoxicity. The decreased cytotoxicity was due to the surface passivation of nAg by d‐sorbitol. Loading with d‐sorbitol enhanced the dispersity of nAg and maintained its native shape, in contrast to the well‐known l‐cysteine modification. The high dispersity and native shape of nanomaterials are critical to their applications. The antimicrobial effects of nAg‐sorbitol were more persistent than those of naked nAg for both G^−^ and G^+^. Screening small metabolites from cells as nanomaterial functional coatings can simultaneously improve the biocompatibility and functionality of nAg and could also be applied to other nanomaterials.

## 4 Experimental Section

*Nanomaterial Characterization*: The nAg (production number XFJ14) used was obtained from Nanjing XFNANO Materials Tech Co., Ltd., Nanjing, China. The characterization of nAg included observation by a transmission electron microscope (TEM, JEM‐2100F, Japan) and a scanning electron microscope (SEM, JSM‐7800, Japan) and the acquisition of the lattice pattern by X‐ray diffraction (XRD, Ultima IV, Japan). The characterization of the metabolite/nAg complex is detailed below.

*Cell Culture and Screening of Metabolites*: The human hepatocyte line LO_2_ (Shanghai Gefan Biotechnology. Co., Ltd) was cultured in Roswell Park Memorial Institute (RPMI) 1640 medium supplemented with fetal bovine serum (FBS, 10%) and antibiotics (penicillin and streptomycin, 1%). The viability of LO_2_ was determined by the 3‐(4,5‐dimethylthiazol‐2‐yl)‐2,5‐diphenyl tetrazolium bromide (MTT) assay. To determine a suitable concentration of nAg for toxicological analysis, different concentrations of nAg (10, 25, 50, 75, and 100 mg L^−1^) were added to the culture medium for 24 h, followed by the MTT assay with an automatic microplate reader (Bio Tek, USA). Cells were seeded in 6‐well microplates and nAg was added at the screening concentration (100 mg L^−1^) for the metabolic assay. The method for the cell metabolic assay was described in our previous study.^65^ Briefly, intracellular metabolites were extracted from cells by liquid–liquid extraction and subjected to nitrogen blow‐off, lyophilization, and subsequent analysis by gas chromatography coupled to quadruple mass spectrometry (GC‐MS, 6890N/1965973, Agilent, USA). The relative contents of metabolites identified by GC‐MS were visualized by a heat map using MetaboAnalyst 3.6. The distance measure and clustering algorithm of the heat map were analyzed by Euclidean and Ward, respectively. To obtain critical small metabolites, the identified metabolites were quantified by the external standard method and screened with statistical analyses including volcano plots (*t*‐tests and fold change analysis, fold change >1.4 and *p* < 0.05), PLS‐DA according to the variable importance in projection scores and SAM (delta = 1.7).

*Effects of Metabolites on nAg Cytotoxicity*: The cell viability of LO_2_ was determined by MTT and apoptosis assays. After cultivation in the presence of nAg (100 mg L^−1^) for 24 h, cells were washed three times with phosphate buffer saline (PBS), and the metabolites (i.e., l‐aspartic acid, citric acid, malic acid, d‐sorbitol, and myoinositol) selected by screening from the 54 identified metabolites were added to the medium. After 12 h of cultivation, cells were washed for MTT analysis by an automatic microplate reader (Bio Tek, USA) or digested for apoptosis analysis by a flow cytometer (FCM, BD LSRFortessa, USA). The amounts of nAg uptake and changes in cell size were also detected by FCM, and the data were analyzed with FlowJo 10. The oxidative stress induced by nAg was probed with 2′,7′‐dichlorodihydrofluorescein diacetate (DCFH‐DA) by using an automatic microplate reader (Bio Tek, USA), as described in our previous study.^65^

*Characterization of Metabolite/nAg Complex*: The size distribution and zeta potential of the metabolite/nAg complexes were examined with a ZETAPALS/BI 200SM instrument (Brookhaven Instruments Corporation, USA). The pH values of nAg suspension (30 mg L^−1^) mixed with metabolites at 1, 10, 100, 1000, and 10 000 mg L^−1^ were determined by using pH test papers (pH 1–14, Shanghai, SSS Reagent Co., Ltd). The nanocomplex of the optimal metabolite (d‐sorbitol) with nAg was further characterized by XPS (Axis Ultra DLD, UK), and the data were analyzed by CasaXPS.

*Quantification of Dissolved Ag^+^*: The nanocomplexes of the metabolites at 100 mg L^−1^ and nAg at 30 mg L^−1^ in ultrapure water were placed in an incubator (37 °C) for 12 h. The nAg was separated from Ag^+^ by centrifuging 5 mL of the sample at 5000 × *g* through 3 kDa ultrafiltration tubes (j‐1427, Millipore, USA) for 30 min. The Ag in the filtrate was acidified (1% HNO_3_) and analyzed by inductively coupled plasma mass spectrometry (ICP‐MS, Elan DRC‐e, USA).

*Adsorption of Metabolites on nAg*: Identical amounts (1 mg L^−1^) of d‐sorbitol and myoinositol were individually added to nAg suspension (30 mg L^−1^), and the mixtures were incubated for 12 h, followed by centrifugation at 10 000 × *g* for 20 min. The supernatant was collected, blown off, lyophilized, and analyzed by GC‐MS (6890N/1965973, Agilent, USA).

*Measurement of Ag^+^ and nAg in Cells*: As nAg was specifically enriched in Triton X‐114, AgNPs and Ag^+^ could be separated by separating the Triton X‐114 phase and the aqueous phase.^66^, ^67^ The addition of Na_2_S_2_O_3_ simultaneously chelated Ag^+^ to avoid its coextraction with nAg.^68^ The dissolved Ag^+^ and nAg could be isolated by a cloud point extraction method. Briefly, cells in 6‐well microplates were treated with nAg (100 mg L^−1^) for 24 h and with optimized d‐sorbitol (770, 77, 7.7 ng per well) for 12 h. After complete washing with PBS, the cells were collected and lyzed, then mixed with 9 mL of HNO_3_ (pH 3.4) supplemented with 0.1 mL of Na_2_S_2_O_3_ (1 m) and 0.2 mL of Triton TX‐114 (10% w/v). The mixture was incubated on ice for 20 min and then transferred to a water bath (40 °C) for 30 min, followed by centrifugation at 5000 × *g* for 20 min to facilitate phase separation. The two phases (nAg in the TX‐114 phase and Ag^+^ in the aqueous phase) were separately digested and quantified by ICP‐MS (Elan DRC‐e, USA).

*Effects of D‐Sorbitol on Ag^+^ Cytotoxicity*: After 24 h of cultivation with AgNO_3_ (10 mg L^−1^), cells were washed and cultured in media containing d‐sorbitol (77, 7.7, 0.77 ng per well) for another 12 h. The cell death and oxidative stress induced by Ag^+^ were detected by the MTT assay and DCFH‐DA probes, respectively. The effects of d‐sorbitol on Ag^+^ were investigated by adding d‐sorbitol (1, 10, and 100 mg L^−1^) to AgNO_3_ solution (30 mg L^−1^), followed by ultrafiltration (3 kDa at 5000 × *g* for 30 min) and ICP‐MS (Elan DRC‐e, USA) detection.

*Effect of D‐Sorbitol on Metabolism with nAg Exposure*: Cells seeded in 6‐well plates were first treated with nAg (24 h) and then d‐sorbitol (770, 77, and 7.7 ng per well) was added. After 12 h treatment, cells were lyzed and metabolites were liquid–liquid extracted with chloroform, methanol, and ultrapure water, following by derivatization and analysis with GC‐MS (6890N/1965973, Agilent, USA). The metabolic analysis was same to the above metabolomics analysis.

*Synthesis and Characterization of nAg‐Sorbitol and nAg‐Cysteine*: nAg‐sorbitol and nAg‐cysteine were prepared by mixing nAg (3 mg mL^−1^) with d‐sorbitol (50 × 10^−3^ m) and l‐cysteine (50 × 10^−3^ m), respectively. To completely wrap d‐sorbitol on nAg and make nanomaterial biocompatible for cells, the concentrations of used d‐sorbitol for the synthesis of nAg‐sorbitol were optimized to obtain the maximal adsorption mass on nAg (over 6 mg per g nAg). The mixture was vigorously stirred for 24 h in darkness and washed with ultrapure water. The products were freeze‐dried and stored in darkness. Both nanomaterials at the same concentration (30 mg L^−1^) were allowed to stand for 24 h, and the released Ag^+^ was quantified by ICP‐MS (Elan DRC‐e, USA) after ultracentrifugation in a 3 kDa ultrafiltration tube. The size distribution and zeta potential of the synthesized nanomaterials were characterized by a ZETAPALS/BI 200SM instrument (Brookhaven Instruments Corporation, USA), the morphology by TEM (Hitachi HT7700, Japan) and an SEM (JSM‐7800, Japan), and the elemental composition by XPS (Thermo Scientific ESCALAB 250Xi, USA).

*Cytotoxicity of nAg‐Sorbitol and nAg‐Cysteine*: Cells were treated with nAg, nAg‐sorbitol, and nAg‐cysteine (100 mg L^−1^) for 24 h, followed by MTT assays and ROS detection, as described above.

*Antibacterial Tests: E. coli* (ATCC 25922, Miaoling, China) and *S. aureus* (ATCC 25923, Solarbio, China) were used in the antibacterial tests. Bacteria were transferred from liquid Luria–Bertani (LB) medium to 96‐well microplates containing AgNO_3_ (100 mg L^−1^), nAg (200 mg L^−1^) or nAg‐sorbitol (200 mg L^−1^), and the increase in OD at 600 nm was detected with an automatic microplate reader (Bio Tek, USA). The OD values after 0, 6, 9, 12, and 24 h were recorded. In addition, d‐sorbitol (1000, 100, and 10 mg L^−1^) and nAg (200 mg L^−1^) were added separately and antibacterial effects after 12 and 24 h were determined by detecting OD increase. Bacterial proliferation tests were performed by using PI‐SYBR green I double staining with FCM (488 nm excitation wavelength, Accuri C6 Plus, BD, Singapore) at 12 h. The shapes and size of the bacteria were also measured by FCM, and the data were analyzed with BD CSampler Plus, USA.

*Statistical Analyses*: At least three replicates were performed in each test. One‐way analysis of variance (ANOVA) was performed in IBM SPSS Statistics 21, and *p* < 0.05 indicated statistical significance. The metabolic analyses, including metabolic visualization (i.e., heat map), volcano plots, PLS‐DA, and SAM were performed in MetaboAnalyst 3.6.

## Conflict of Interest

The authors declare no conflict of interest.

## Supporting information

Supplementary
