# Supplementary material for: Screening Small Metabolites from Cells as Multifunctional Coatings Simultaneously Improves Nanomaterial Biocompatibility and Functionality
Source: Adv Sci (Weinh). 2018 May 23;5(7):1800341. doi: 10.1002/advs.201800341 (PMC6051401; doi:10.1002/advs.201800341)
Supplement: Supplementary file 1 — Supplementary [file ADVS-5-1800341-s001.pdf]

Copyright WILEY-VCH Verlag GmbH & Co. KGaA, 69469 Weinheim, Germany, 2018.

ADVANCED SCIENCE
Open Access

Supporting Information

for *Adv. Sci.,* DOI: 10.1002/advs.201800341

Screening Small Metabolites from Cells as Multifunctional Coatings Simultaneously Improves Nanomaterial Biocompatibility and Functionality

*Anqi Sun, Zhan Ban, Li Mu, and Xiangang Hu**

Supporting Information

Screening Small Metabolites from Cells as Multifunctional Coatings Simultaneously Improves Nanomaterial Biocompatibility and Functionality

*Anqi Sun, Zhan Ban, Li Mu and Xiangang Hu**

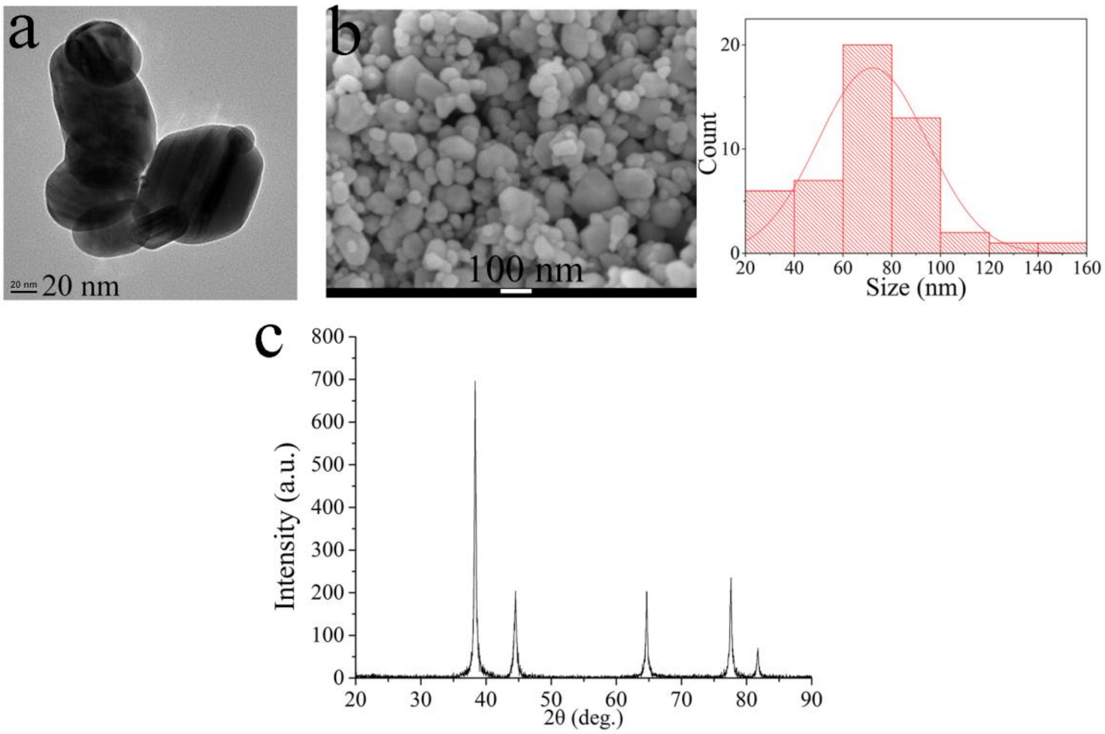

Figure S1. Characterizations of nAg: a) TEM image of nAg; b) SEM image of nAg; c) XRD pattern of nAg.

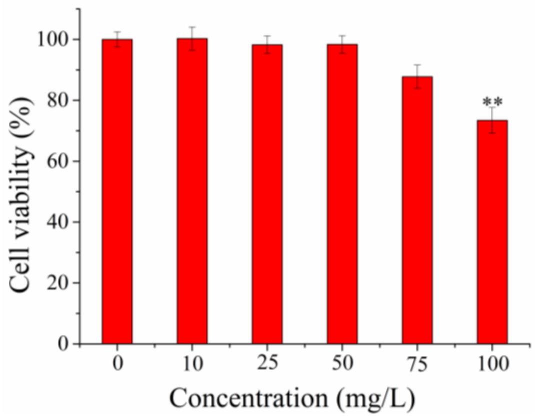

Figure S2. Effects of nAg on cell viability. "**" suggests p<0.01 compared with the control.

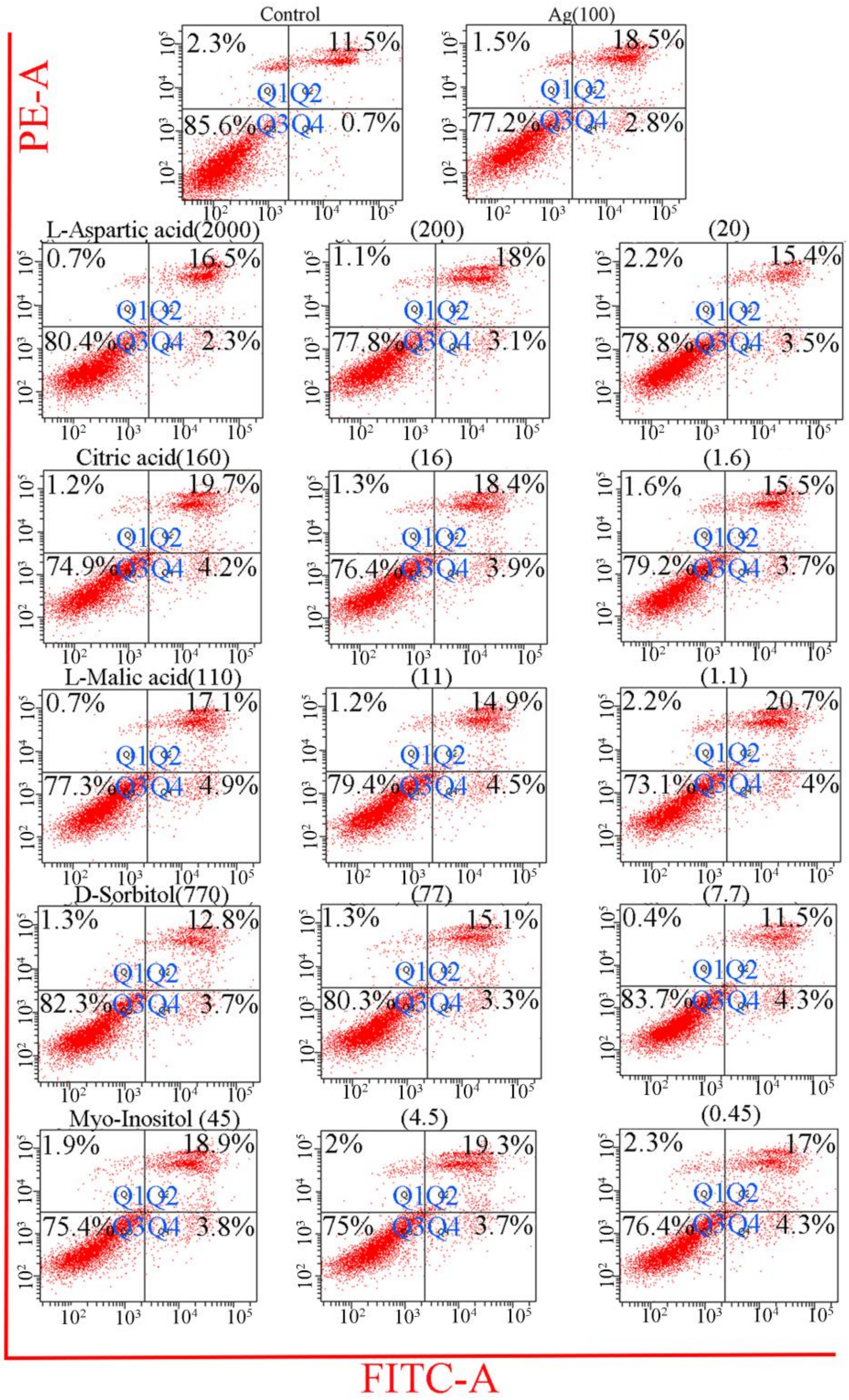

Figure S3. Cell apoptosis. “Ag (100)” indicates the groups treated with nAg at 100 mg/L. The numbers after metabolite names are the concentrations of added metabolites (ng/10^6 cells). Q1, Q2, Q3 and Q4 indicate dead cells, cells in late apoptosis, viable cells and cells in early apoptosis, respectively.

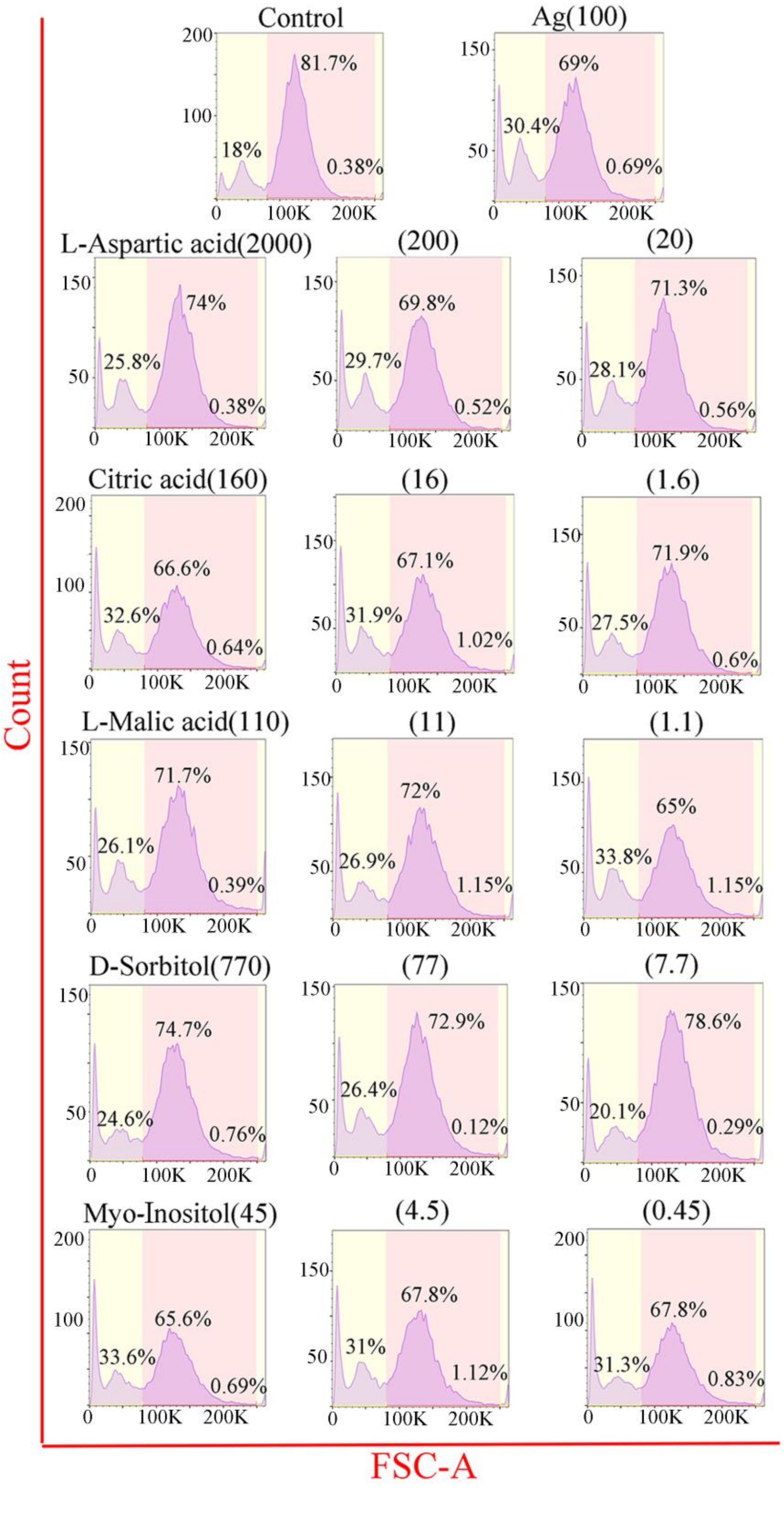

Figure S4. Size change of cells. "Ag (100)" indicates groups treated with nAg at 100 mg/L. The numbers after metabolite names are the concentrations of added metabolites (ng/10^6 cells).

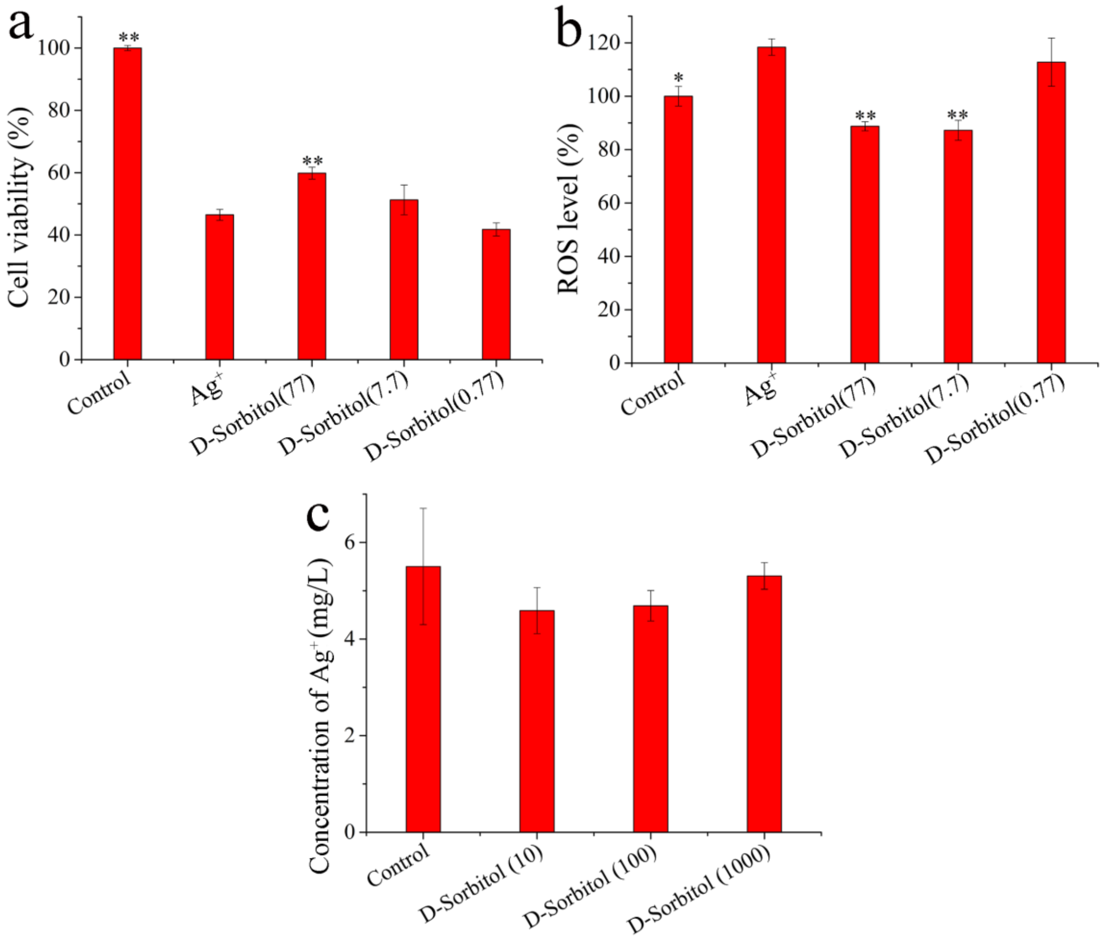

Figure S5. Effects of D-sorbitol on cytotoxicity of Ag^+: a) cell viability assay; b) ROS level of cells; c) effects of D-sorbitol on the contents of dissolved Ag^+. "*" and "**" suggests $p<0.05$ and $p<0.01$ compared with cells adding $AgNO_3$ (10 mg/L), respectively. The numbers after "D-Sorbitol" in Figure S5a and S5b are the concentrations of added metabolites (ng/10^5 cells). The numbers after "D-Sorbitol" in Figure S5c are the concentrations of metabolites (mg/L).

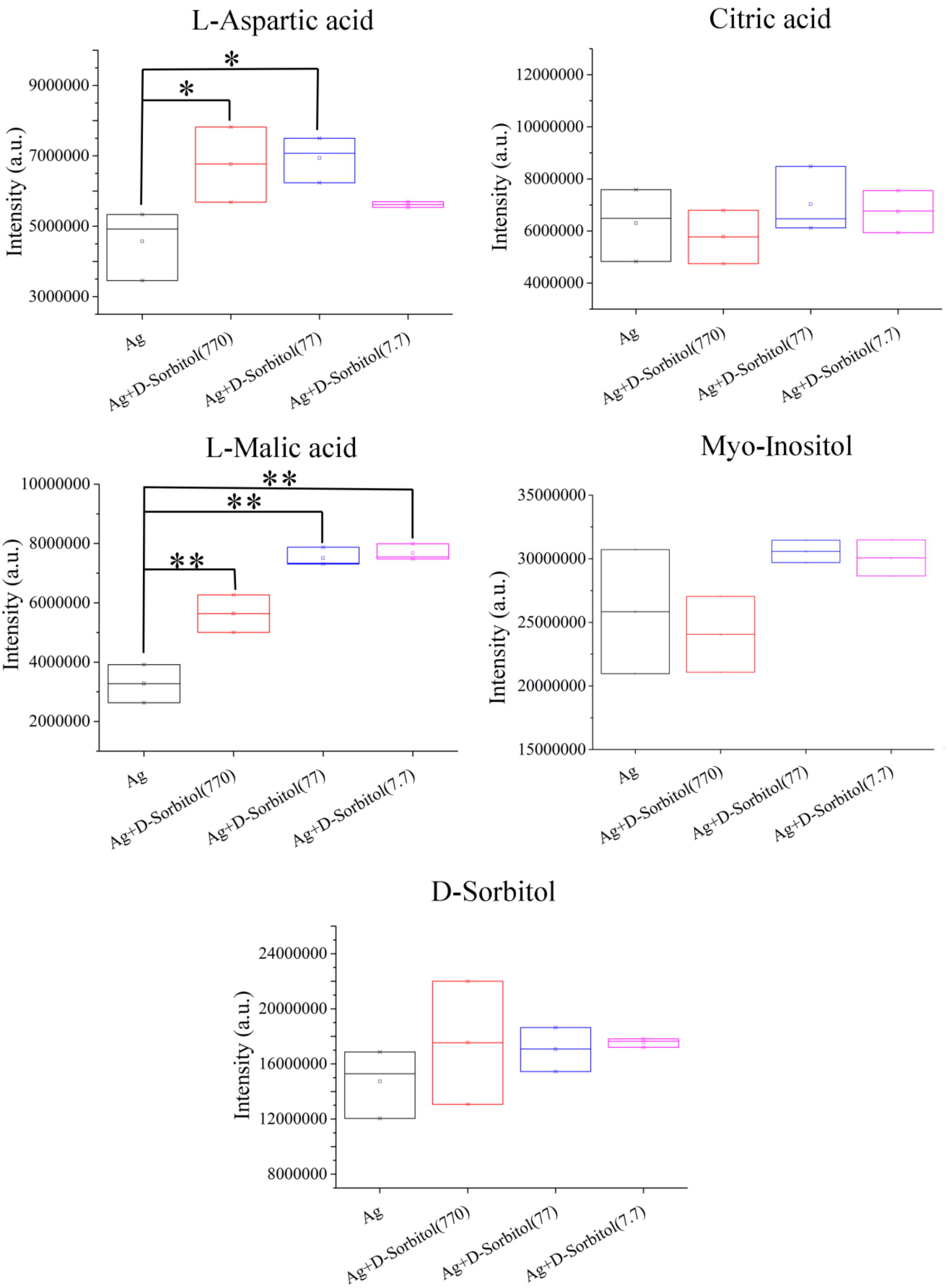

Figure S6. Effects of D-sorbitol on metabolism. The numbers after metabolite names are the amount of added metabolites (ng/10^6 cells). "*" and "**" indicate $p < 0.05$ and $p < 0.01$, respectively.

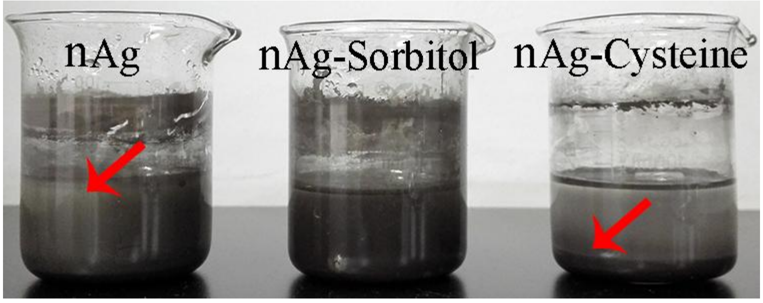

Figure S7. Digital images of the synthesized nAg-based nanomaterials. The red arrows denote the aggregations.

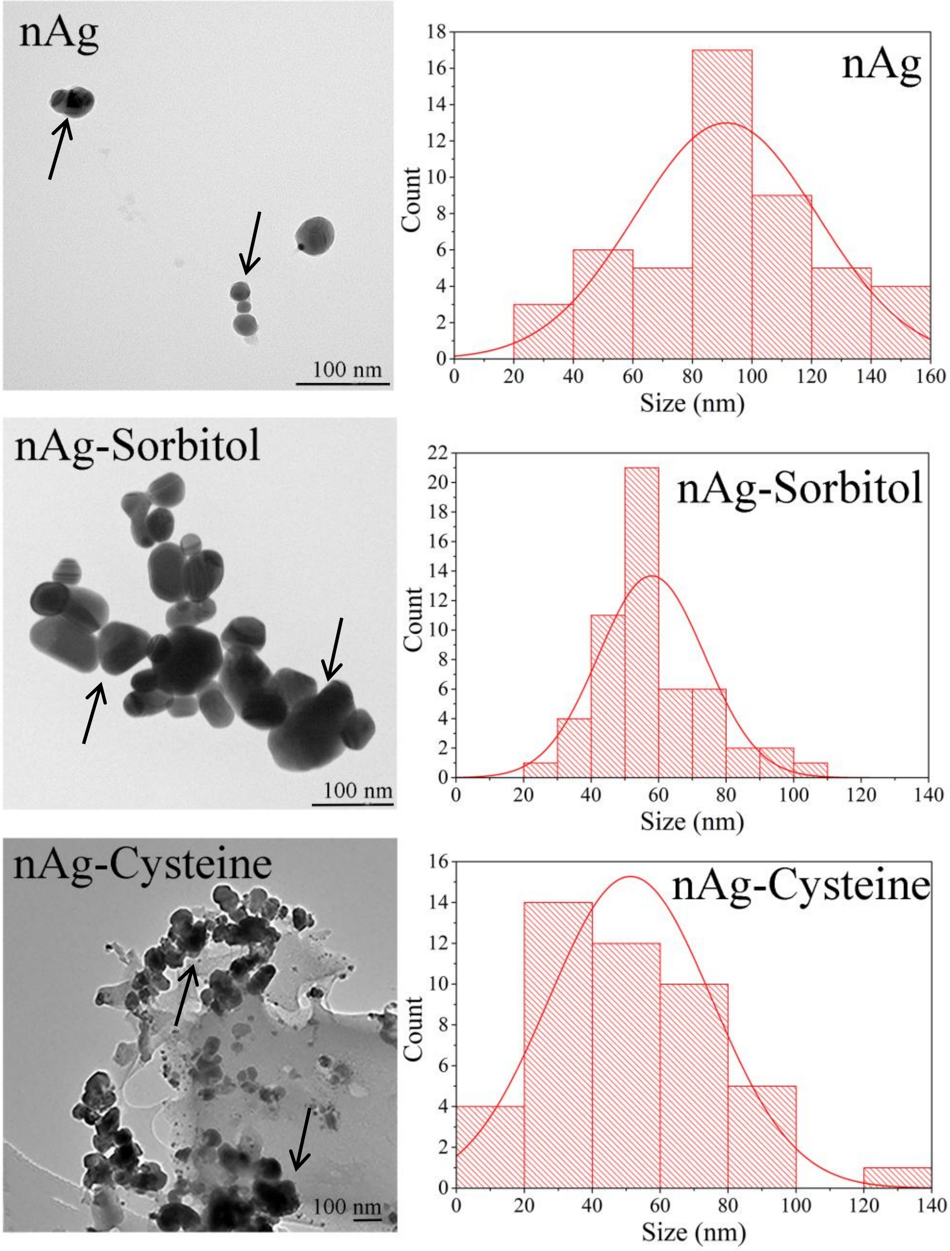

Figure S8. Low-magnification TEM images of nAg, nAg-sorbitol and nAg-cysteine and the corresponding size distribution curves. The arrows denote the tested nanoparticles.

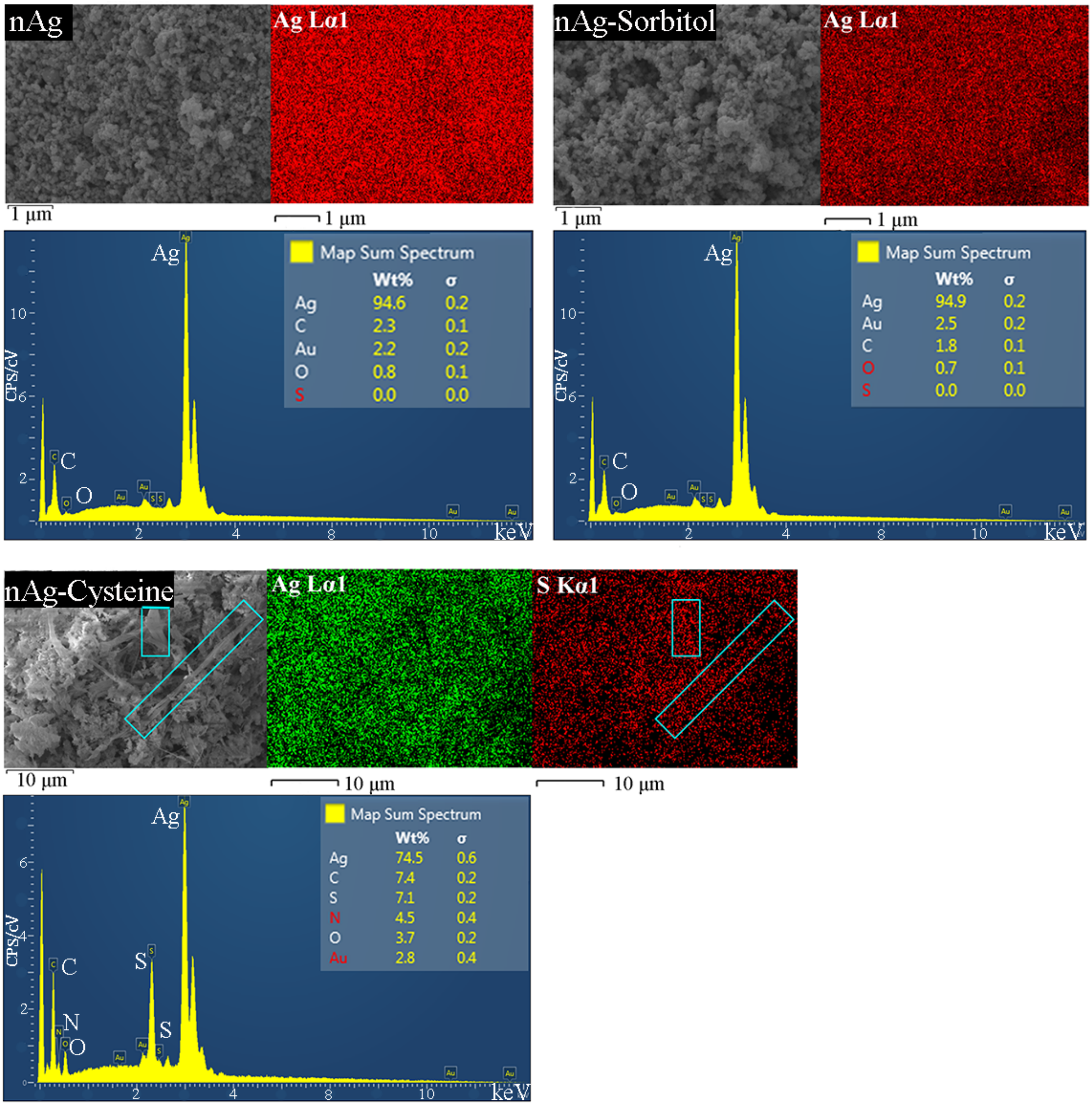

Figure S9. Characterization of nAg, nAg-sorbitol and nAg-cysteine by EDS mapping.

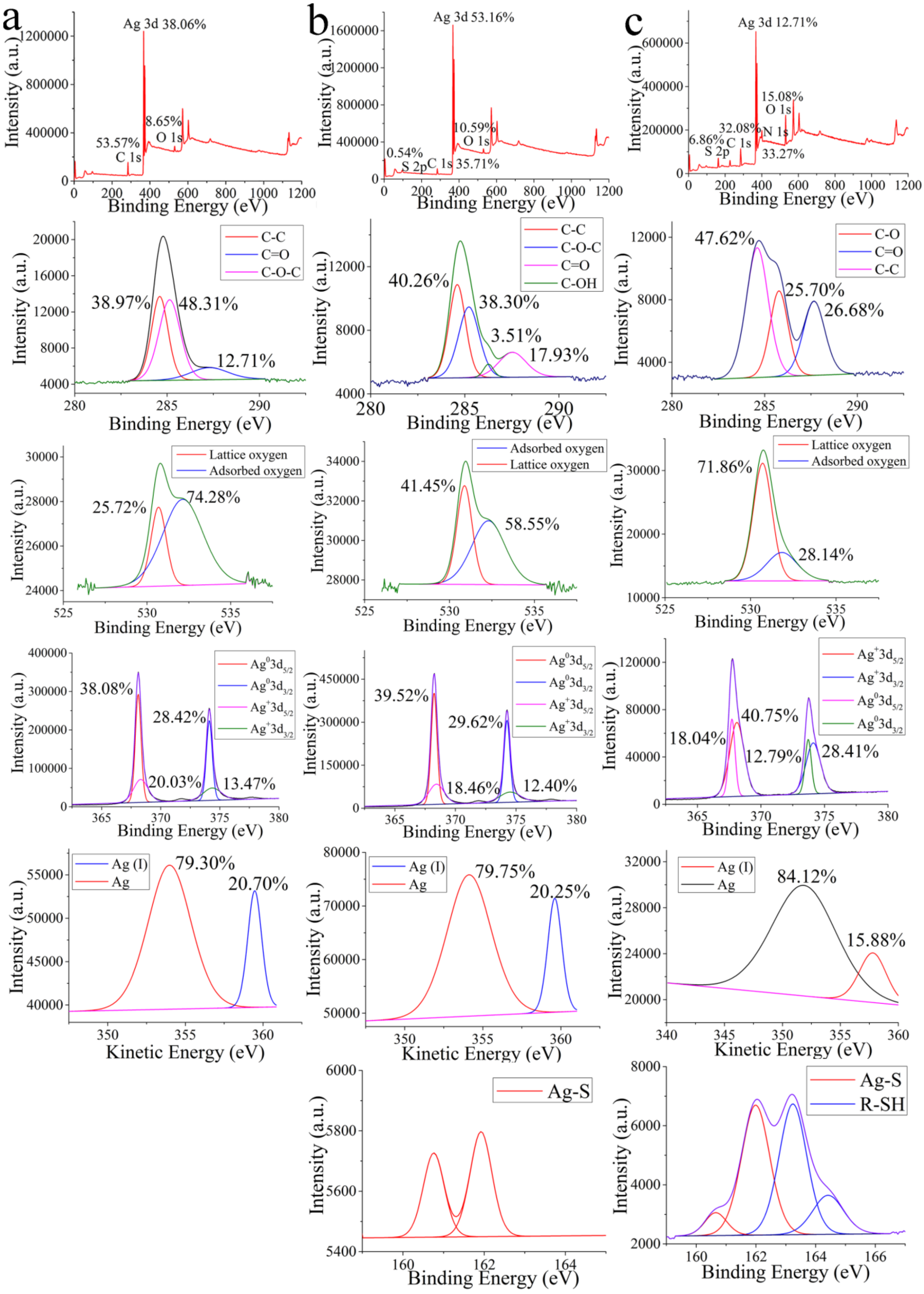

Figure S10. XPS spectra of nAg-based nanomaterials: a) XPS spectra of nAg; b) XPS spectra of nAg-sorbitol; c) XPS spectra of nAg-cysteine.

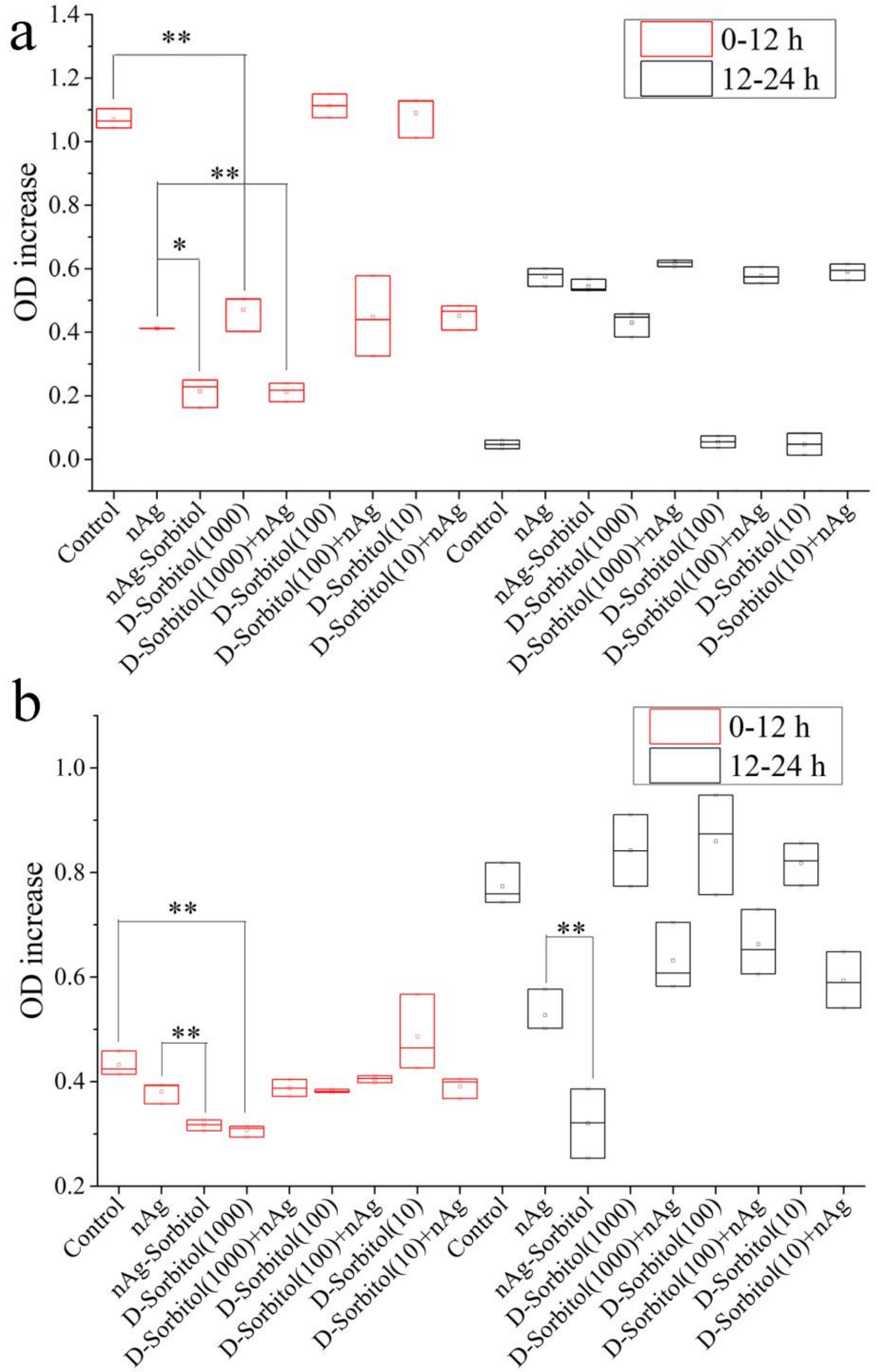

Figure S11. Antibacterial effects of nAg, nAg-sorbitol, D-sorbitol and complex of D-sorbitol and nAg: a, Time-dependent OD changes of *E. coli*; b, time-dependent OD changes of *S. aureus*. "*" and "**" indicate $p < 0.05$ and $p < 0.01$, respectively.

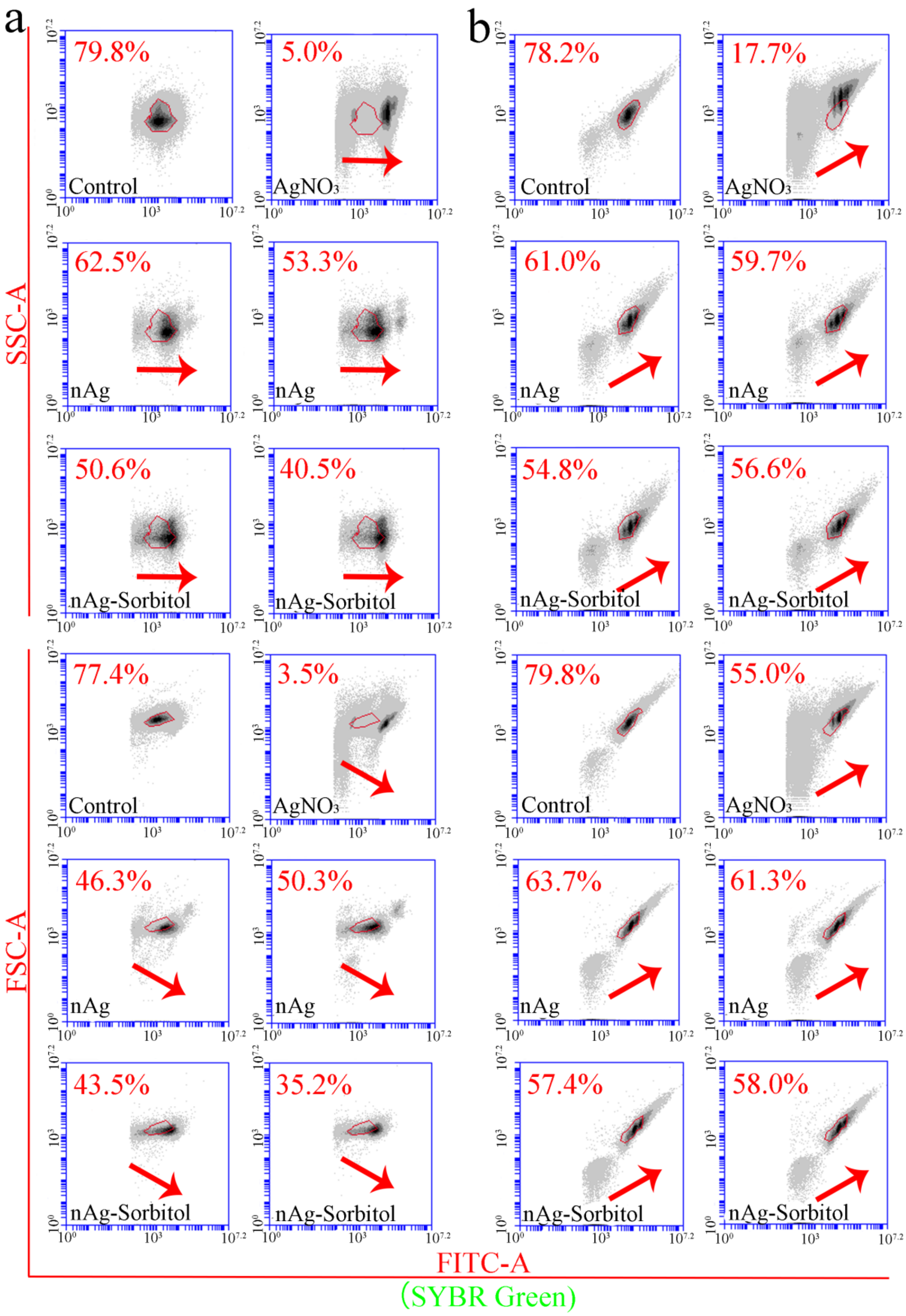

Figure S12. Biocidal effects of Ag^{+} and nAg-based nanomaterials on bacteria with SYBR Green staining: a) biocidal effects on *E.coli*; b) biocidal effects on *S. aureus*; The red arrows denote the shift tendency of the cell population.

Table S1. Metabolites screened by volcano plot

Metabolite name	Fold change	*p* value
L-Valine	0.11250	0.033950
L-Cysteine	0.14248	0.0022153
DL-Ornithine	0.24656	0.028600
Stearic acid	0.83086	0.041392
Myo-Inositol	1.4327	0.0033456
Malic acid	1.8358	0.0022505
Citric acid	1.9264	0.030741
D-Sorbitol	4.9388	0.0033886
L-Aspartic acid	133.65	0.00033815

Table S2. Metabolites screened by SAM

Metabolite name	*d* value	StDev	*p* value	*q* value
L-Aspartic acid	-11.396	98152	0.0018519	0.075409
Malic acid	-6.9511	12837	0.0055556	0.075409
Myo-Inositol	-6.2479	16791	0.0074074	0.075409
D-Sorbitol	-6.2263	303510	0.0092593	0.075409
Citric acid	-3.2717	49844	0.024074	0.14005
L-Cysteine	6.9804	7270.5	0.0037037	0.075409

Table S3. Metabolites screened by PLS-DA

Metabolite name	comp 1	comp 2	comp 3	comp 4	comp 5
L-Aspartic acid	-0.45243	0	0	0	0
Malic acid	-0.40778	0	0	0	0
Myo-Inositol	-0.39170	0	0	0	0
D-Sorbitol	-0.39113	0	0	0	0
Citric acid	-0.20666	0	0	0	0
Sucrose	-0.056478	0	0	0	0
Phenylalanine	0	0	0	0	-0.65888
Glycine	0	0	0	0	-0.58175
L-Isoleucine	0	0	0	0	-0.27790
Nonanoic acid	0	0	-0.029973	0	-0.19566
Glycolic acid	0	0	-0.029973	0	-0.19566
5-Oxoproline	0	0	-0.16660	0	-0.14303
Leucine	0	0	0	-0.35918	0
L-Tyrosine	0	0	-0.18556	-0.13961	0
D-Mannopyranose	0	0	0	-0.076339	0
Glycerol monostearate	0	0	-0.71378	0	0
1-Monopalmitin	0	0	-0.56998	0	0
D-Lactose	0	0	-0.16593	0	0
N-.alpha.-Acetyl-L-Lysine	0	-0.50319	0	0	0
Phosphonic acid	0	-0.46818	0	0	0
Palmitic Acid	0	-0.41743	0	0	0

L-Threonine	0	-0.20350	0	0	0
L-Hydroxyproline	0	0	0	0	0
D--Galactose	0	0	0	0	0
d-Mannose	0	0	0	0	0
Asparagine	0	0	0	0	0
L-Lysine	0	0	0	0	0
Serine	0	0	0	0	0
3-.alpha.-Mannobiose	0	0	0	0	0
D--Turanose	0	0	0	0	0
Maltose	0	0	0	0	0
Arachidic acid	0	0	0	0	0
2-Palmitoylglycerol	0	0	0	0	0
D-Glucose	0	0.094240	0	0	0
Glucose oxime	0	0.29306	0	0	0
2-Butenedioic acid	0	0.29306	0	0	0
Urea	0	0.29306	0	0	0
D--Talose	0	-0.080170	0.060856	0	0
D-erythro-2-Pentulose	0	0	0.15320	0	0
D-Trehalose	0	0	0.15320	0	0
D-Gluconic acid	0	0	0.15320	0	0
Lactic Acid	0	0	0	0.13851	0
Pentaric acid	0	0	0	0.20118	0
Cholesterol	0	0	0	0.26699	0
L-Glutamic acid	0	0	0	0.48663	0
Propanoic acid	0	0	0	0.52837	0
L-Serine	0	0	0	-0.27364	0.010402
D-Allose	0	0	0	0.35195	0.054373
.beta.-Alanine	0	-0.19746	0	0	0.12797
Phosphoric acid	0	0	0	0	0.18374
Stearic acid	0.16138	0	0	0	0
L-Valine	0.19233	0	0	0	0
DL-Ornithine	0.21662	0	0	0	0
L-Cysteine	0.40835	0	0	0	0

Table S4. Metabolites screened by volcano plot, SAM and PLS-DA

Metabolite name	Volcano plot	SAM	PLS-DA
L-Valine	+	-	+
L-Cysteine	+	+	+
DL-Ornithine	+	-	+
Stearic acid	+	-	+
Myo-Inositol	+	+	+
L-Malic acid	+	+	+
Citric acid	+	+	+
D-Sorbitol	+	+	+
L-Aspartic acid	+	+	+
Sucrose	-	-	+

"+" means metabolites successfully screened by the corresponding screening method while "-" illustrates metabolites failing to screened by the method.

Table S5. Quantification of the five screened metabolites

Metabolite name	Control ($ng/10^6$ cells)	nAg (100 mg/L) ($ng/10^6$ cells)	Decrease/added contents ($ng/10^6$ cells)
L-Aspartic acid	207.758±31.339	<detection limit	207.758±31.339
Citric acid	33.447±8.105	17.363±2.611	16.084±5.494
Malic acid	19.519±2.098	8.635±2.774	10.884±0.676
D-Sorbitol	99.705±31.422	22.905±17.182	76.800±14.240
Myo-Inositol	14.738±1.068	10.287±0.617	4.451±0.451
